# Persistent high major adverse cardiac outcome of 7% with chronic total occlusion intervention in patients with stable coronary artery disease

**DOI:** 10.1007/s12471-026-02017-x

**Published:** 2026-01-22

**Authors:** Mohammad Reza Movahed

**Affiliations:** 1 Department of Medicine, University of Arizona Sarver Heart Center, 85724 Tucson, AZ USA; 2 Department of Medicine, University of Arizona College of Medicine Phoenix, Phoenix, AZ USA

Dear Editor,

In the manuscript “Longitudinal results from a dedicated chronic total coronary occlusions percutaneous coronary intervention program” [1], the authors reported improvement in major adverse cardiac outcome (MACE) from 13% to 7% with percutaneous coronary intervention (PCI) of patients with chronic total occlusion (CTO). They called it a successful program. However, despite being a dedicated CTO center, this MACE rate is still too high, consistent with our recent publication [2], and is downplayed in this manuscript. It is important to elaborate that CTO, based on the definition, is a stable coronary artery disease (CAD) and a stable CAD has a MACE rate of about 1.1 to 2.4% a year [3]. It is important to emphasize that CTO intervention is still associated with a much higher MACE rate than the natural occurrence of MACE rate in stable CAD (per definition, including CTO). Therefore, it should only be performed in patients with resistant severe angina despite maximal medical therapy who are aware of the high-risk intervention without any long-term mortality benefit, and it is done for symptom relief [3].
